# Exploring organisational mechanisms in PRO-based follow-up in routine outpatient care - an interpretive description of the clinician perspective

**DOI:** 10.1186/s12913-018-3352-y

**Published:** 2018-07-13

**Authors:** Caroline Trillingsgaard Mejdahl, Liv Marit Valen Schougaard, Niels Henrik Hjollund, Erik Riiskjær, Kirsten Lomborg

**Affiliations:** 1 Department of Public Health, Aarhus University, Bartholins Allé 2, DK-8000 Aarhus, C Denmark; 2 The Research Program in Patient Involvement, Aarhus University Hospital, Palle Juul-Jensens Boulevard 99, DK-8200 Aarhus, N Denmark; 3 WestChronic, Occupational Medicine, University Research Clinic, Aarhus University, Herning Gl Landevej 61, DK-7400 Herning, Denmark; 4 Department of Clinical Epidemiology, Aarhus University Hospital, Aarhus, Olof Palmes Allé 43-45, DK-8200 Aarhus, N Denmark; 5 DEFACTUM, Social & Health Services and Labour Market, Central Denmark Region, Olof Palmes Allé 15, DK-8200 Aarhus, N Denmark; 6 Department of Clinical Medicine, Aarhus University, Palle Juul-Jensens Boulevard 82, DK-8200 Aarhus, N Denmark

**Keywords:** Patient-reported outcome (PRO) measures, Outpatient follow-up, Qualitative research, Interpretive description, Organisational mechanisms, Customisation

## Abstract

**Background:**

Patient-reported outcome (PRO)-based follow-up is a new model of service delivery, where PRO measures are used as the very basis for demand-driven outpatient follow-up in patients with chronic diseases. Adopting the clinicians’ perspective, we aimed to explore what happens when PRO-based follow-up is implemented in routine clinical practice. We also aimed to identify organisational mechanisms related to PRO-based follow-up.

**Methods:**

The methodological approach of this interview study is interpretive description, informed by a perspective of critical realism. Semi-structured interviews were conducted with 13 clinicians (eight nurses and five physicians) working with PRO-based follow-up in outpatient care for epilepsy in the Central Denmark Region.

**Results:**

PRO-based follow-up gave rise to ambivalence in clinicians. Seen from the clinicians’ perspective, PRO-based follow-up could both increase and decrease the quality of follow-up. Moreover, PRO-based follow-up both enhanced and impaired clinicians’ work experiences. Additionally, the clinicians used strategies to ease some of the perceived disadvantages. The clinicians did extra tasks and worked around the scope of PRO-based follow-up. Thus, clinicians constituted a professional buffer as they deflected some of the negative mechanisms associated with PRO-based follow-up.

**Conclusions:**

As a model of a service delivery, PRO-based follow-up is highly dependent on the clinicians’ day-to-day management of the system, and mechanisms related to routine use of PRO measures in outpatient follow-up are complex. Paying attention to the organisational settings is critical for PRO-based follow-up to improve quality of care and enhance patient-centred care.

**Electronic supplementary material:**

The online version of this article (10.1186/s12913-018-3352-y) contains supplementary material, which is available to authorized users.

## Background

Improvement of patients’ health is the overriding goal of healthcare. Until recently, the pathophysiological disease understanding has been key to modern Western medicine’s effort to reach this goal [1]. However, recognition of the importance of taking into account the patient’s perspective has gained prominence in recent years; in particular in the context of efforts to improve quality and effectiveness of healthcare [1, 2]. Since patients’ perspective on their disease can only be given by themselves, this paradigm shift towards patient involvement has led to global proliferation and application of patient-reported outcome (PRO) measures throughout the healthcare system [1]. Patient involvement refers particularly to patients’ right to have a central position in their own healthcare, and the benefits of this are expected to be a better patient outcome as a result of an improved interaction between the patient and the clinician [3].Thus, to day patients are more empowered, call for more involvement and their contribution is valued by healthcare authorities [4, 5].

In this study, we explored nurses’ and physicians’ experiences with the routine use of PRO measures in clinical practice in three outpatient clinics in the Central Denmark Region. The study reported here is part of a larger study of PRO-based follow-up in epilepsy outpatient care.

A patient-reported outcome measure is defined as “a measurement based on a report that comes directly from the patient about the status of a patient’s health condition without amendment or interpretation of the patient’s response by a clinician or anyone else” [6]. In broad terms, PRO measures are questionnaires that measure patients’ perception of the impact of a condition and its treatment on their health [7] Thus, PRO measures are useful when measuring aspects that are best known to patients or best measured from the patients’ perspectives [1]. Such aspects include, e.g., symptoms not obvious to observers, psychological symptoms, the frequency and severity of symptoms, the impact of disease on daily life, social wellbeing, cognitive functioning, role activities, and health-related quality of life [8]. Traditionally, PRO measures have been used at a group level in research and quality improvement [2]. However, focus on use of PRO measures at the individual patient level has increased during recent decades [9]. The use of PRO measures in clinical practice has several documented positive effects, including optimal monitoring of treatment response, facilitating patient-centred communication, support of the clinical decision-making process, optimal efficient use of healthcare resources, reporting of adverse drug reactions and as a tool for hospital performance assessment [10–15]. Thus, PRO measures represent an idea to which is ascribed a range of positive effect on both patient and organisational level.

In 2016, Danish Regions decided to expand the positive experiences with PRO measures as a general idea. The aim was to improve the quality of health care by spreading knowledge about PRO measures and to standardise questionnaires on a national basis [16]. Thus, PRO systems are now being implemented at scale, based on national initiatives [17–19]. However, in previous research, it was recognised that PRO-measures are not a self-acting mechanism that automatically enhance quality in clinical practice [20, 21]. In order for the potential of PRO measures to be realised, the idea of using PRO measures must be adopted by all relevant members of the organisation.

We know from institutional theory that new ideas are not automatically and uncritically adopted and incorporated into organisations [22]. They are translated, shaped and changed during implementation processes, and formed by both supporting and inhibiting powers and mechanisms [23, 24]. Thus, organisations can enact a range of strategies in response to pressures toward adopting new ideas [22].

In routine outpatient practice, nurses along with physicians are the front-line clinicians responsible for the practical application of PRO-based follow-up [25]. Among clinicians, there seems to be diverging attitudes towards the use of PRO measures in clinical practice [9, 26, 27]. Some strongly advocate their use in clinical practice, as they believe that PRO measures provide data that make a valuable contribution to clinical practice; others, however, are sceptical [2, 9, 27]. These diverging attitudes led us to consider that the perspectives of front-line clinicians as end-users of PRO measures can contribute to identifying organisational mechanisms that influence the success or failure of PRO-based follow-up. We also find it valuable to explore if these mechanisms have lasting potential when PRO measures are implemented at scale in the future.

The aim of this study is to explore what happens when PRO-based follow-up is implemented in routine clinical practice. We aimed to identify organisational mechanisms in relation to PRO-based follow-up seen from the clinicians’ perspective.

## Methods

### Design

The methodological approach used in this interview study was interpretive description (ID) [28], informed by a perspective of critical realism [29].

ID is an applied, inductive research strategy emphasising the significance of performing research arising from clinical practice with the aim of improving this practice [28]. As required for interpretive description studies, we gathered and analysed data concurrently, as we allowed the preliminary analysis to guide the subsequent focus in the data collection phase. This iterative process of data collection and analysis allowed us to compare, reflect upon and explore data elements throughout the process. [28].

We chose to integrate tenets of critical realism into our ID strategy as we aimed to frame, identify and comprehend complex mechanisms of action in relation to clinicians’ experiences with PRO-based follow-up. Critical realism rests on a belief in the existence of three different layers of reality. These three ontological domains encompass the “real”, the “actual” and the “empirical” [29, 30]. Accordingly, reality is not transparent; however, it has powers and mechanisms that are not directly observable but can be experienced only indirectly by their ability to cause events in the empirical domain [29].

Both ID and critical realism influenced the development of the interview guides and the analyses of the empirical data. We found that this integration of ID and a critical realist ontology gave us a sufficient strategy for understanding the complex mechanisms underlying the organisational implementation of the idea of PRO measures in clinical practice.

### Setting

AmbuFlex is a generic web-based PRO system that supports demand-driven outpatient follow-up as opposed to follow-up with regular fixed consultation. AmbuFlex was developed by the third author and is the frontend of the WestChronic system, used for research purposes in clinical epidemiological studies science 2004 [10]. AmbuFlex is not limited to specific patient groups and as per April 2018, AmbuFlex was implemented in 21 diagnosis groups in ten hospitals in Denmark [19]. AmbuFlex’s overall aims are to improve the quality of care, enhance the patient-centredness of care and reallocate healthcare resources by using PRO measures as the basis for follow-up [10, 25]. The method is termed PRO-based follow-up and differs from the traditional clinical use of PRO measures, where PRO measures simply compose a supplement to the consultation at each follow-up visit. PRO-based follow-up represents a new model of service delivery where the patient’s PRO measures are used as the very basis for outpatient follow-up.

In 2012, AmbuFlex/Epilepsy was implemented in three different outpatient clinics in the Central Denmark Region, and it is now the standard follow-up for 54% of patients with epilepsy in these clinics [25] (Table 1).

**Table 1:** Outpatients with epilepsy in PRO-based follow-up

	Aarhus	Holstebro	Viborg	Total
Epilepsy patients in total January 2018, *n*	3958^a^	1305^a^	899^a^	6162
Current AmbuFlex/epilepsy patients January 2018, *n* (%)	2499 (63)	579 (44)	264 (29)	3342 (54)

Outpatients with epilepsy and referral of patients to PRO-based follow-up (AmbuFlex/epilepsy) in Aarhus University Hospital, Holstebro Regional Hospital and Viborg Regional Hospital. ^a^Numbers based on information provided by the outpatient clinics

Prior to implementing AmbuFlex, follow-up for patients with epilepsy was managed by regular pre-scheduled visits, typical every 6th or 12th month. These visits may have occurred when the patient was well, and neither the patient nor the clinician regarded the visit necessary [25, 31]. In AmbuFlex/Epilepsy, regularly scheduled follow-ups may be substituted by regular questionnaires filled out by patients at home. The follow-up activity is determined by a clinician and patients receive the PRO questionnaire at fixed intervals (3, 6, or 12 months). Patients who are not capable or willing to fill in the PRO questionnaire on the Web have the opportunity to fill in the questionnaire on paper [10].

Clinicians use the patients’ self-reported PRO data as a decision aid to identify those who need clinical attention [31]. Nurses or physicians refer patients to PRO-based follow-up after having assessed their health status and their ability to fill in PRO questionnaires.

The questionnaire includes information about frequency of seizures, wellbeing, symptoms, health-related quality of life and information specific to aspects of daily life with epilepsy (Additional file 1). The PRO questionnaire was developed in close cooperation with clinicians. After a pilot test, PRO-based follow-up was implemented, and experiences with the system are continuously evaluated. Items are revised in an ongoing iterative process in which clinicians have influence in improving the system. Clinicians receive no formal training in practicing PRO-based follow-up [25].

The patients’ PRO responses are automatically processed according to a specific algorithm and given a “green”, “yellow” or “red” status. A red status indicates that the patient needs or wishes contact, a yellow status indicates that the patient may need contact, and a green status indicates that the patient has no current need or wish of attention. A green status is automatically handled by the server software and a new PRO assessment is scheduled, whereas a clinician has to decide whether a yellow status patient needs contact or not.

In all cases, patients can request a contact, either a phone call or a consultation in the outpatient clinic, which will automatically overrule any automated decision that no visit is needed. Non-responders get three reminders and are contacted if they do not respond. Clinicians keep track of incoming yellow and red responses, and of non-responders [25]. The response rate is estimated to be 92% for the initial PRO questionnaire and 95% for the subsequent ones [25].

The PRO overview [25] is presented graphically to the clinician within the electronic health record system. The PRO algorithm and the PRO overview are used as decision aids together with other available health record information to inform the decision whether the patient needs a contact or not [25]. The nurses in the epilepsy outpatient clinics are responsible for handling the patients’ red and yellow PRO responses. In case of a red response, the nurse assesses whether the patient should be called in for consultation in the outpatient clinic or be contacted by phone. For yellow responses, the nurse assesses whether the specific patient needs a contact or not.

If the nurse assesses the patient’s PRO data to be without health issues that need clinical attention, the patient will receive a new questionnaire at a scheduled point in time. In addition, the nurse also decides whether the contact or consultation should be with a nurse or a physician. When handling PRO responses, nurses can refer tasks to physicians electronically, e.g. checking blood test, making phone calls to patients and authorising medication changes. Table 2 presents an overview of the distribution of green, yellow and red PRO response in the three epilepsy outpatient clinics. A total of 13,995 follow-up contacts in the 7-year period were PRO-based.

**Table 2:** PRO-based contacts in outpatients with epilepsy in Aarhus University Hospital, Holstebro Regional Hospital and Viborg Regional Hospital 2011–2018.

	Aarhus	Holstebro	Viborg	Total
PRO questionnaire responses in total from 2011 to 2018, *n* (%)	11,588 (100)	1893 (100)	514 (100)	13,995 (100)
PRO-algorithm, *n* (%)				
Green	1501 (13)	413 (22)	94 (18)	2008 (14)
Yellow	7307 (63)	1114 (59)	344 (67)	8765 (63)
Red	2780 (24)	366 (19)	76 (15)	3222 (23)
No Contact^a^, *n* (%)	5456 (47)	1290 (68)	343 (67)	7089 (51)
Contact^b^, *n* (%)	6071 (52)	569 (30)	156 (30)	6796 (49)
Pending^c^, *n* (%)	61 (0.5)	34 (2)	15 (3)	110 (1)

^a^No Contact: Responses without need of additional clinical contact.

^b^Contact: Responses requiring additional clinical contact (telephone call or consultation in outpatient clinic).

^c^Pending: responses pending a clinical assessment

### Sampling of informants and data collection

The inclusion criteria for interviews were physicians and nurses working with AmbuFlex/epilepsy in the Central Denmark Region who had at least 6 months of experience with AmbuFlex. All of the 13 clinicians who were invited to participate in the study agreed to be interviewed. Thus, they were sampled entirely by convenience, as the 13 clinicians were the only clinicians who met the inclusions criteria in our data collection period (Table 3).

**Table 3:** Clinician participant profile

		No. 13 (%)
Profession	Nurse	8 (62)
	Physician	5 (38)
Gender	Female	10 (77)
	Male	3 (23)
Hospital	Holstebro	2 (15)
	Viborg	4 (31)
	Aarhus	7 (54)
Experience with PRO-based follow-up (months)	6–12	2 (15)
	13–24	1 (8)
	25–36	3 (23)
	> 36	7 (54)

The interviews were conducted by the first author from May 2016 to June 2017 and were carried out in offices of the outpatient clinics. Based on the research question and an initial literature review, an interview guide was developed. Preliminary analysis of data from the first interviews were used to develop the questions for the subsequent data collection. During the interviews, the clinicians were first invited to share their personal experiences of PRO-based follow-up. Subsequently, five main themes were raised during the interviews: PRO-based follow-up’s influence on: a) work procedures in the outpatient clinic b) intra- and interdisciplinary cooperation, c) the quality of the outpatient follow-up d) the patient-clinician relation and e) professional competencies and identity.

### Data analysis

All interviews were audio-recorded with the clinicians’ permission. They were transcribed verbatim by the first author, and each participant was given a code number. The first and last author collaborated on the analysis, supported by discussion with co-authors. In the analysis, we first immersed into the data by reading all of the transcripts in order to develop a sense of the whole beyond our immediate impression of the data material. Thereafter, we arranged the data in terms of patterns that seemed to reflect similar properties. This was an ongoing iterative process in which patterns were gathered and disassembled repeatedly.

As we organised the data in thematic groups, specific dimensions of clinician experiences were increasingly gathered in recurring themes. These tentative groupings led us to consider the patterns and variety within those groups across the whole material. As the analysis process developed, possible relationships between the groups of data became more apparent, and we conceptualised the findings by extracting thematic patterns that represent clinician experiences with PRO-based follow-up. Data management was facilitated by the qualitative software programme NVivo™ [32].

In the presentation of our findings, the term clinicians is used when the theme concerns both nurses and physicians, whereas for themes that apply only to one of the professions, the specific profession will be stated explicitly.

## Results

We found that overall the clinicians were ambivalent towards PRO-based follow-up. They emphasised that it could both increase and decrease the quality of the follow-up. They also emphasised that PRO-based follow-up both enhanced and impaired their work experiences. Moreover, we found that clinicians used strategies to remedy some of the perceived disadvantages in PRO-based follow-up. Table 4 illustrates the themes and subthemes.

**Table 4:** Themes and subthemes

Themes	Subthemes
The impact of PRO-based follow-up on quality of outpatient care	Improvement experiences
	Deterioration experiences
The impact of PRO-based follow-up on work experiences	Job enrichment
	Organisational challenges
	Being overburdened
	Ethical issues
Clinicians constituting a professional buffer	Doing extra tasks
	Customising patient contact

### The impact of PRO-based follow-up on quality of outpatient care

#### Improvement experiences

Clinicians found that a patient’s repeatedly collected PRO measures improved the clinicians’ overview and assessment of the patient’s health status over time. They emphasised that AmbuFlex supported demand-driven outpatient follow-up implying that the patients with the greatest need of attention did, in fact, receive this attention. Furthermore, clinicians believed that patients benefitted from filling in the questionnaire and they were convinced that the patients’ capacity for self-management rose owing to PRO-based follow-up. They emphasised that PRO questionnaires may support the patients’ learning process, making the patients more competent. Thus, the clinicians were convinced that PRO-based follow-up overall increased the quality of the outpatient follow-up. One physician said:

*It’s a good way to structure my time; it allows me to take care of the ones who need me the most (...) That’s the main thing for me. But what’s really important is that it simply qualifies the contacts.*

Due to the incorporated safety net in the PRO system, clinicians found that it prompted better patient safety as the system would alert if a patient refrained from answering. Clinicians emphasised that PRO measures in relation to psychosocial values send a signal to the patients that the clinicians found that these topics were important. Thus, clinicians found that PRO gave rise to growing awareness of psychosocial problems and problems in relation the patient’s everyday life. Clinicians found that PRO-based follow-up supported patient-centred outpatient follow-up, as PRO guided them to focus on the problems that were most importance to the patient.

Nurses emphasised that PRO-based follow-up had yielded a refined nursing care, as the nursing assignments and responsibility embedded in the system made nursing more visible and important. Due to the questions about psychosocial problems, the nurses experienced assignments to be aligned with the “core of nursing”. As one nurse put it:

*Thus, nursing care becomes more visible when we use the AmbuFlex questionnaire correctly, I think. We now have a better understanding of the areas where we have something to contribute (...) All those soft areas, or all those things related to living an everyday life where you experience a loss of control, and where the people around you also need to adapt. Here, the nurse has knowledge she can contribute with.*

Additionally, nurses found that their nursing practice improved, given that the PRO questionnaire guided the dialogue with the patient during telephone conversations. PRO measures made it easier to prioritise conversation topics, and PRO measures enhanced discussions of sensitive issues with the patient. Nurses found that PRO made it easier for some patients to initiate dialogues concerning sensitive problems like impotence or anxiety.

Thus, clinicians were convinced that PRO-based follow-up underpinned a better, more patient-centred and safer healthcare service.

#### Deterioration experiences

Some clinicians expressed suspicion towards the trustworthiness and value of the patients’ self-assessments. Clinicians found that some patients were incapable of making the right assessment of their health status. The clinicians had concerns regarding the patients’ capability to reflect on the questions and fill in the questionnaire relevantly, as they regarded patients’ reflection and immersion as a prerequisite to efficient and safe PRO-based follow-up.

In addition, clinicians emphasised that the questionnaire made it easier for patients to lie about their health status; especially when it came to the existence of seizures and whether they had driven a car during the past month. These clinicians found that the distance between the patient and the clinician induced by PRO-based follow-up reduced the relevance of the patient’s answers.

Several nurses found that PRO-based follow-up restricted their nursing practice. They thought of the questionnaire as simplistic, rigid and impersonal. They emphasised that “something was missing” due to the distance between them and the patients. The nurses described how their assessment of the patients via the questionnaire or based on a telephone conversation were lacking several dimensions. Often the nurses would have a hard time describing what was missing, but most of them found that they lacked the possibility to use more senses, and they emphasised that they could not use their intuition as they did not meet the patient face-to-face. The nurses were often “looking for the person” behind the questionnaire. They made extra efforts to get a grip of the patient, as they found that the questionnaire did not provide enough information. As one nurse put it:

*It is the tradition I was brought up with! It’s the issue of; well, its really important to include the patient’s view. It’s so important that I can feel, sense, hear and then make an assessment of the situation as a human and a professional (...) I think that the sense for the patient that you sometimes intuitive have may now be lost because I don’t meet the patient (...). It definitely is somewhat more difficult in a questionnaire and on the phone; “Well, is it even appropriate for me to ask this?” These things are harder to assess on the phone; “Can I be as direct as I would if we met face to face?” So, some of the questions that I would put to them if we were in the same room, I may not ask on the phone.*

All nurses found that PRO-based follow-up impaired the relationship between them and the patients. Nurses emphasised that this was a significant loss that could decrease the quality of the nursing care. The nurses thought of the lack of interpersonal contact as a loss for both the patients and themselves personally. Furthermore, the absence of a relationship potentially brought the risk that the nurses might handle their response in a more mechanical manner. Nurses found that there was a risk that nursing became too mechanical in PRO-based follow-up and argued that when they had to handle a high number of responses, it could be difficult to keep remembering that there was a real person behind each questionnaire. The questionnaires all looked alike and handling many responses was exhausting. One nurse emphasised:

*If your whole day goes by providing 15 responses and no other tasks, then that’s a lot of responses to analyse based on a questionnaire. Therefore, it’s really important to stay aware that there’s a real person in the other end. This is not just a questionnaire I’m reviewing; it is a real flesh and blood human who provided these answers. And maintaining that focus can be difficult if you have too many tasks and the list just keeps on growing and growing, then the focus may suffer a bit. When you think, this is really going to be a tough task. And that’s not the idea at all. It NEEDS to be a task that has the same priority as when people attend our consultations.*

Some nurses felt that PRO-based follow-up prevented them from performing “real nursing”, as it introduced a distance between the nurse and the patient. Thus, nurses experienced that the distance between them and the patients reduced the quality in outpatient follow-up.

### The impact of PRO-based follow-up on work experiences

#### Job enrichment

The certainty that they were offering patients a better and safer follow-up than previously gave rise to feelings of increased professional satisfaction among the clinicians as did also the fact that they were convinced that PRO-based follow-up facilitated a more appropriate prioritisation of patients. Addressing this issue, one nurse said:

*It (AmbuFlex) makes sense, I think. And it gives you a sense of satisfaction because I think we make it easier for the patient in this way.*

Due to a changed pattern of inter-professional work procedures, nurses experienced an enhanced feeling of responsibility and reported positive experiences of having more independent assignments. Thus, PRO-based follow-up seems to enhance the nurses’ professional autonomy as it increased their possibilities to make independent choices and gave them the freedom to choose between various actions. Especially, handling the patients’ PRO questionnaires and assessing their need for contact were assessed positively. In addition, nurses found that the altered work assignments and shift in responsibility produced by PRO-based follow-up allowed them to feel more recognised and respected by the physicians. This gave them a feeling of increased personal and professional satisfaction.

#### Organisational challenges

In general, clinicians experienced organisational challenges in relation to PRO-based follow-up. Overall, they emphasised that PRO-based follow-up had increased their workload and significantly changed work procedures. The responsibilities related to PRO-based follow-up undertaken by clinicians were perceived as additional to their existing professional responsibilities. Consequently, they expressed the view that PRO-based follow-up added to their workload. As one physician put it:

*We have shifted from seeing the patients right here in the outpatient clinic to calling them on the phone. The consequence is that phone contacts have simply exploded, and our phone consultations are long, just as long as the visits in the clinic. So this has moved people from the chair to the phone.*

Another physician emphasised:

*Well, I think that it’s important that you don’t believe that this will solve all issues related to waiting lists and such stuff, because I’m under the impression that hospital managers and that kind of people think that they have found the pot of gold at the end of the rainbow (laughs). That it’ll sort of solve all our problems (…). I mean, it’s an upgrade of the patient pathway, meaning that it boosts quality, but it doesn’t necessarily save us any time or work.*

Clinicians emphasised that the amount of telephone conversations with patients had increased substantially. They found that the work procedures had changed in the outpatient clinics, as the nurses referred more task electronically to physicians than previously when all patients attended the outpatient clinic for follow-up visits regularly. Physicians emphasised that the system had significantly increased administrative assignments with no direct patient contact. Furthermore, physicians experienced an increased workload in general in the outpatient clinic caused by PRO-based follow-up because the nurses had more independent work assignments and could no longer help out the way they did before. For example, one physician said:

*In the past, the nurses helped us out a lot with the consultations, they don’t do that at all anymore because now they attend to their own patient procedures. And that means that they are putting into this huge efforts that maybe they didn't have quite the same chance to contribute with in the past when they also had to order blood samples and help out more, they don’t really do that much any more. At least to a much lesser extent.*

Furthermore, clinicians sometimes experienced a mismatch between ideal PRO-based follow-up and actual practice. E.g. due to organisational circumstances and conditions in the outpatient clinics, it was not always possible to handle the responses within 1–2 weeks. The clinicians considered unacceptable giving responses given after 10–14 days. These late responses were a cause of concern among nurses and physicians alike.

Overall, clinicians problematised that PRO-based follow-up had been implemented in the organisation without releasing extra financial resources to handle the PRO responses and respond to the increased complexity of the visiting patients’ medical problems.

#### Being overburdened

Clinicians emphasised that the increased workload occasionally resulted in experiences of being overburdened. Both nurses and physicians emphasised that the increase in telephone consultations could be both exhausting and demanding. In addition, physicians experienced an increased pressure from the nurses who needed support to decide if the patient needed attention or not.

The physicians all acknowledged that the nurses’ need for supervision was reasonable. However, they at times found themselves overwhelmed with work associated with PRO-based follow-up. Furthermore, physicians pointed out that it produced an increased complexity in the remaining patients in the outpatient clinic. Due to the fact, that the PRO system facilitates a demand-driven outpatient follow-up, the physicians all experienced an increase in the complexity of the visiting patients’ medical problems. There were no “easy patients” anymore. One physician stated:

*The typical hello and goodbye patients have disappeared, largely. And that can be somewhat burdensome, because a monster problem walks in the door with every single patient (...) I mean, I’m at my whit’s end every time I see a patient: How am I supposed to solve that? (laughs) I mean, it keeps me on my toes constantly, it’s not relaxing or comfortable. No, I’m professionally challenged when the patients show up.*

Some physicians found that this increase in the complexity was exhausting and that it challenged them professionally. The increased complexity had a negative bearing on some of the physicians’ job satisfaction. In periods with busyness, PRO-based follow-up was often perceived as a stress factor. Clinicians emphasised that the demands induced by PRO-based follow-up often exceed their available resources.

Given that it was a nursing task to handle responses, and the fact that the number of response fluctuated caused most of the nurses to be constantly aware of the PRO alert list. In other words, PRO-based follow-up was perceived as an endless string of work assignments. The fact that the nurses never knew how many responses there were on the list led to increased negative thoughts about their job when they were off work, for example during weekends and holidays. One nurse expressed this as follows:

*This may also be stressful. Will I be able to complete this on time? All the time it’s there in the back of my head; I really need to check up and respond. Today, I’m down to two (responses on the list) and I feel great because I know things are under control and I can be proactive. I simply can’t when there are 122 patients on the list.*

Another nurse, who had experienced being the only nurse working with PRO-based follow-up said:

*I don’t mind saying that it’s been stressful for me, let there be no doubt about that. Because, no time has been set aside for this, I’ve been the only one there, in the epilepsy outpatient clinic. And no time had been allocated to AmbuFlex, and I just had no way of getting it done. Sometimes, there were lots of work, and I’d feel guilty, because I hadn’t a chance to look at some of the questionnaires (...) Because no-one else looked into these things when I was on vacation, if I fell or took a day off. Because I was the only one working AmbuFlex. So nothing was done, when I was not there.*

A nurse emphasised that when PRO-based follow-up stressed her, she made poorer decisions; she would, for example, prioritise the patients with least problems on the list just to reduce the numbers more quickly, and she tended to make fewer clarifying phone call to patients when she was in doubt as to whether they needed attention or not. Given that handling the responses is organised as a flexible assignment that the nurses can do whenever they can find time for it during working hours, the handling of the incoming questionnaires tended to be deprioritised in favour of other assignments. The nurses often seized the opportunity when working evening or night shifts in other wards to complete some of their responses during late hours. In this manner, working with and thinking of AmbuFlex was an omnipresent factor for the nurses.

Thus, both nurses and physicians found that they at times had a hard time keeping up their spirits. They occasionally felt overburdened and exhausted by PRO-based follow-up.

#### Ethical issues

On one hand, clinicians emphasised that PRO-based follow-up could entail feelings of guilty conscience towards the patients. It caused concern among clinicians, as they at times worried that some patients were “lost” in this system. They were afraid that they might disregard some important issues that the patients refrain from reporting for some reason. Nurses emphasised that PRO-based follow-up at times gave rise to ethical dilemmas. For example, when the patients had explicitly noted that they would not accept a phone call regarding their answer and the nurse suspected some serious health issue based on their assessment of the patient’s PRO.

On the other hand, the clinicians emphasised that PRO-based follow-up could ease their conscience. They realised that due to lack of economic resources in healthcare, it would be impossible to schedule routine consultations for all. Therefore, sending the patients questionnaires and giving them the opportunity to choose contact was perceived as an act consolation. One physician expressed it this way:

*It seems fair to say that AmbuFlex may help us feel less guilty. Because when we think, whether it’s real or not, but when we think that we don’t have enough time to solve our tasks, and therefore need to cut down on some things (...) Well, now, thanks to the questionnaire, we have some knowledge about the patient, and the patient has had the opportunity to let us know if they want a consultation or not, and then somehow it may be said that we’re not to blame if something goes wrong. So in a way it may ease our conscience about not having enough time for patients.*

Thus, with PRO-based follow-up, the physicians found that they could load some of the responsibility for the patient’s well-being onto the patients and that eased their conscience.

### Clinicians constituting a professional buffer

We found signs that clinicians used strategies to ease some of the perceived disadvantages. The clinicians did extra tasks and worked around the scope of PRO-based follow-up. Thus, clinicians constituted a professional buffer as they deflected some of the negative mechanisms associated with PRO-based follow-up.

#### Doing extra tasks

In order to keep patients feeling safe and confident, clinicians took on extra actions that were beyond the scope of PRO-based follow-up. Nurses would, for example, make extra phone calls to elderly or vulnerable patients to reassure them that the outpatient clinic had received their questionnaire. One nurse said:

*But I do call many on my own accord, I do (...) I may call elderly patients who have completed the questionnaire for the first time just to let them know that we have seen their answers. Just to let them know that it all works, right? And I think that’s time well spent, it does make them feel more secure, right. I think that means a lot.*

Sometimes the nurses would check “green answers” and call the patients because they knew from experience that very few patients had that low a number of problems, and therefore they were afraid that several green answers in a row might be a mistake. Thus, nurses tried to minimise the risk that the automatic algorithm led to important health issues being disregarded.

#### Customising patient contact

In handling “yellow” answers, nurses often challenged the patient’s assessment of need for contact. If the patient had noted “no need for contact”, and the nurse suspected a health issue that needed attention, she would call the patient to ensure that “everything was all right”. The nurses based their behaviour on a professional assessment, which was a mix of specialist knowledge, experience and intuition. One nurse said:

*But I also think it may be dangerous because I experience that some patients do not think about the questions or about how they answer the questionnaire, and then... I worry that the answers we receive may not be useful to us? Then I might just decide to call the patients one more time to be absolutely sure.*

In addition, some nurses also challenged the patient’s assessment of need for contact if the patient had specifically requested a consultation in the outpatient clinic. The nurses would often call the patient and ask why the patient wanted a contact. The nurses justified this in two ways: to target the consultation to the right profession (nurse or physician) and to ensure that the patient actually needed a consultation. More nurses expressed that often it turned out that a telephone conversation was enough, that the patient did not need a psychical consultation and that there was no need to burden the physicians. Thus, nurses sometimes worked around the scope of PRO-based follow-up in order to compensate for the lack of resources in the outpatient clinics.

## Discussion

The findings of this study provide insight into front-line clinicians’ perspectives on PRO-based follow-up in clinical practice. PRO-based follow-up gave rise to ambivalence in clinicians. Seen from the clinicians’ perspective, PRO-based follow-up could both increase and decrease the quality of follow-up. Moreover, PRO-based follow-up both enhanced and impaired clinicians’ work experiences. Additionally, the clinicians used strategies to ease some of the perceived disadvantages.

Some of the organisational mechanisms identified mirror those reported in other studies on clinicians’ experiences with PRO measures in clinical practice. A systematic review of clinicians’ use of PRO measures to improve the quality of healthcare documented that an increased workload was a barrier [26]. A survey of clinicians’ preferences and perceived barriers for routine assessment of PRO measures in paediatric oncology practice illustrated that clinicians strongly value the routine use of PRO measures in clinical practice [27]. However, it was found that the integration of PRO measures in the organisation was limited due to barriers such as lack of time, lack of financial resources and PRO measures not fitting within the existing clinical workflows [27]. These barriers are in line with the clinicians’ experiences of organisational challenges and being overburdened by PRO-based follow-up. Nevertheless, none of these existing reports on clinicians’ experiences with PRO measures addresses clinicians’ experiences with PRO-based follow-up. Some of the clinicians’ perceived barriers in our study related to the distance between them and the patient embedded in PRO-based follow-up. PRO-based follow-up represents substantially changed outpatient care that encompasses components related to the practice of “telecare”. Especially nurses were ambivalent toward providing the care at a distance embedded in PRO-based follow-up. On the one hand, nurses found that their nursing practice improved when the PRO questionnaire guided the dialog with the patients during telephone conversations. On the other hand, nurses found that the distance induced by PRO-based follow-up deteriorated the quality of patient care. Thus, for nurses, providing care at a distance was PRO measures in outpatient follow-up often experienced as problematic. Such concerns are in line with common clinical concerns in relation to telecare [33–35] Nurses are said to have particular concerns about telecare practices, because telecare will impede their relationship with patients and potentially prevent nurses from noticing important signs of trouble [36]. Another important finding in our study was that the clinicians used strategies to ease some of the perceived disadvantages. The clinicians did extra tasks and worked around the scope of PRO-based follow-up. Thus, clinicians constituted a professional buffer as they deflected some of the negative mechanisms associated with PRO-based follow-up. As Table 2 illustrates, the clinicians’ individual assessments and contacts are extensive and therefore serve as a significant supplement to the automated server algorithm build into PRO-based follow-up. Clinicians serving as a professional buffer seems to counteract the built-in standardisation in PRO measures in order to make good the lack of resources in the outpatient clinics. These buffer actions were motivated and guided by the clinicians’ professional ethics and standards, as the nurses and physicians experienced a discordance between the freedom of responsibility that the PRO-system offered and their ethical professional standards. This finding is in line with ideas from institutional research [22–24, 37]. In a theoretical institutional framework, it is stated that institutional ideas must be customised rather than simply adopted by the organisation, as a local customisation of an idea can be decisive for the ability of the idea to function as intended [37]. From a critical realistic viewpoint, the professional buffer serves as an intuitional power making the idea of PRO measures work in routine patient follow-up. Thus, the main mechanism is PRO-based follow-up; yet, it is the professional ethics and standards that make the PRO function as intended. Mechanisms such as workload fluctuations and resource reductions challenge the clinicians’ intentions to provide a more customised and better outpatient care.

Based on our findings and the above discussion, we point to three critical areas when implementing PRO-based follow-up in routine clinical practice. The areas relate to a) the organisation of PRO-based follow-up, b) clinicians, and c) visitation of patients.

### Organisational implications

Our findings documented that clinicians experienced increased workload associated with PRO-based follow-up and being overburdened. We documented that assignments in relation to PRO-based follow-up were given a lower priority in periods of busyness, which resulted in a build-up of PRO responses and a backlog of assignments. An organisational implication may therefore be to adapt clinical settings and by introducing an organisation of work procedures that underpins the prioritisation of PRO-based follow-up assignments on a par with other patient contact tasks. In addition, to comply with the increased workload, resources should be allocated to manage the PRO responses and to adequately handle the increased complexity of the visiting patients’ medical problems. Moreover, we found that both intra- and interdisciplinary support and collaboration in PRO-based follow-up were regarded as very important by clinicians. Therefore, organisation of PRO-based follow-up allowing for collegial feedback and discussions seems important in securing the success of PRO-based follow-up.

### Implications related to clinicians

We documented diverging attitudes among clinicians towards the value of patients’ self-assessments. When clinicians do not appreciate the information provided by the patients’ PRO data and distrust the very value of these assessments, considerations regarding the clinicians’ readiness to work with PRO-based follow-up may be raised. Therefore, it is suggested that an awareness of clinical value of patients’ self-assessments could be part of the clinicians’ introduction to and training in working with PRO-based follow-up. Ideally, all clinicians involved in PRO-based follow-up would trust in the value of PRO measures.

### Implications related to visitation of patients

Our study documented that the clinicians had concerns regarding some patients’ ability to fill in the PRO questionnaire, and they questioned the relevance of some patients’ self-assessments. This raises the question if the present referral approach is inexpedient. We suggest that a strong focus needs to be placed on the quality of the patient’s introduction to PRO-based follow-up. Ideally, decisions regarding the individual patient’s enrolment in PRO-based follow-up should be a shared decision between patient and clinician.

There are some limitations to our study. Concerning the transferability of the study’s findings, we acknowledge that the three involved outpatient clinics were “early adopters” whose self-imposed implementation of PRO-based follow-up may have influenced the identified mechanisms in relation to the ability of PRO measures to improve outpatient care [37].

This study has focused on the clinicians’ perspective on PRO measures in clinical practice. Given that organisational mechanisms unfold at several levels in the healthcare organisations, it would be beneficial to also explore the perspectives of, e.g., patients, clinical leaders and other decision-makers in clinical practice. Thus, further research should include other organisational settings that have not implemented PRO-based follow-up as a self-imposed measure, and the perspectives patients and of other stakeholders should also be explored. Despite its limitations, the study certainly adds to our understanding of the complex organisational mechanisms relating to PRO-based follow-up in clinical practice. Thus, we expect our findings to be of relevance for other contexts in healthcare organisations were PRO measures are being implemented as they shed light on the mechanisms that underpin PRO measures as an instrument to facilitate quality outpatient follow-up.

## Conclusions

PRO-based follow-up is a model of service delivery that is highly dependent on the clinicians’ day-to-day management of the system, and mechanisms relating to the routine use of PRO measures in outpatient follow-up are complex. Clinicians’ independent navigation within the PRO system are of particular significance for the ability of PRO measures to function as intended. Moreover, appropriate visitation of patient to PRO-based follow-up along with a general clinical appreciation of patients’ self-assessments are of critical importance for the success of PRO-based follow-up. Thus, we encourage clinicians and managements to carefully consider and discuss the implications of PRO-based follow-up before applying PRO-based follow-up to clinical practicen.

## Additional file

Additional file 1: AmbuFlex/epilepsy questionnaire. (PDF 822 kb)
