# Supplementary material for: Exploring organisational mechanisms in PRO-based follow-up in routine outpatient care - an interpretive description of the clinician perspective
Source: BMC Health Serv Res. 2018 Jul 13;18:546. doi: 10.1186/s12913-018-3352-y (PMC6044066; doi:10.1186/s12913-018-3352-y)
Supplement: Supplementary file 1 — AmbuFlex/epilepsy questionnaire. (PDF 822 kb) [file 12913_2018_3352_MOESM1_ESM.pdf]

QUESTIONNAIRE

ABOUT YOUR EPILEPSY

When did you have your most recent epileptic seizure? (Write year and month - write "?" if you do not remember)

Year (f. ex. 2011):: ____

Month no. (f. ex. 11): __

How many seizures did you have last year? (if none, write 0)

Number of attacks: : __

How many absence seizures have you had in the last 3 months?

Number of attacks: : __

How many generalized seizures (convulsions) have you had during the last 3 months?

Number of attacks: : __

Answer the following questions only if you have had at least 1 epileptic seizure during the last year

Do your epileptic seizures occur during sleep?	☐ Yes	☐ No	
Are your seizures getting worse?	☐ Yes	☐ No	
Have you sustained an injury during a seizure?	☐ No	☐ Yes, but not a serious one	☐ Serious damage (f. ex. bone fracture, cut wounds)
Have you been in contact with an emergency room because of epilepsy since your last visit to the outpatient department?	☐ Yes	☐ No	

Are your relatives worried about you because of your epilepsy?

Put one tick	☐ Never	☐ Rarely	☐ Occasionally	☐ Frequently	☐ Do not know	☐ Not applicable

During the last 4 weeks to what degree have you suffered from:

Headache	☐ Never	☐ Occasionally	☐ Sometimes	☐ Often	☐ Very often
Dizziness	☐ Never	☐ Occasionally	☐ Sometimes	☐ Often	☐ Very often
Tremor/shacking	☐ Never	☐ Occasionally	☐ Sometimes	☐ Often	☐ Very often
Double vision or other visual disturbances	☐ Never	☐ Occasionally	☐ Sometimes	☐ Often	☐ Very often
Loss of appetite	☐ Never	☐ Occasionally	☐ Sometimes	☐ Often	☐ Very often
Eating too much	☐ Never	☐ Occasionally	☐ Sometimes	☐ Often	☐ Very often
Difficulty remembering	☐ Never	☐ Occasionally	☐ Sometimes	☐ Often	☐ Very often
Difficulty concentrating	☐ Never	☐ Occasionally	☐ Sometimes	☐ Often	☐ Very often
A feeling that you easily become aggressive	☐ Never	☐ Occasionally	☐ Sometimes	☐ Often	☐ very often
Severe fatigue	☐ Never	☐ Occasionally	☐ Sometimes	☐ Often	☐ Very often
Sadness	☐ Never	☐ Occasionally	☐ Sometimes	☐ Often	☐ Very often
Being afraid of having a new seizure during the next weeks	☐ Never	☐ Occasionally	☐ Sometimes	☐ Often	☐ Very often
Lack of interest or pleasure in sexual activity	☐ Never	☐ Occasionally	☐ Sometimes	☐ Often	☐ Very often
Have you in the last 4 weeks had suicidal thoughts?	☐ Never	☐ Occasionally	☐ Sometimes	☐ Often	☐ Very often

Please indicate for each of the five statements which is closest to how you have been feeling over the last two weeks.

Over the last two weeks

I have felt cheerful and in good spirits	☐ All of the time	☐ Most of the time	☐ More than half of the time	☐ Less than half of the time	☐ Some of the time	☐ At no time
I have felt calm and relaxed	☐ All of the time	☐ Most of the time	☐ More than half of the time	☐ Less than half of the time	☐ Some of the time	☐ At no time
I have felt active and vigorous	☐ All of the time	☐ Most of the time	☐ More than half of the time	☐ Less than half of the time	☐ Some of the time	☐ At no time
I woke up feeling fresh and rested	☐ All of the time	☐ Most of the time	☐ More than half of the time	☐ Less than half of the time	☐ Some of the time	☐ At no time
My daily life has been filled with things that interest me	☐ All of the time	☐ Most of the time	☐ More than half of the time	☐ Less than half of the time	☐ Some of the time	☐ At no time

In general, would you say your health is:

Put one tick	☐ Excellent	☐ Very good	☐ Good	☐ Fair	☐ Poor

Compared to one year ago, how would you rate your health in general now?

Put one tick	☐ Much better now than one year ago	☐ Somewhat better now than one year ago	☐ About the same	☐ Somewhat worse now than one year ago	☐ Much worse than one year ago

Do you have other diseases or conditions that have a greater effect on your health than your epilepsy?

☐ Yes

☐ No

The next questions deal with your medical treatment

How often do you think you have forgotten to take some of your medicine?	☐ Daily	☐ Weekly	☐ Monthly	☐ Very rarely, never
Does your epilepsy medicine have side effects?	☐ No	☐ Yes, a few	☐ Yes, some	☐ Yes, many

The next questions deal with work (being a student counts as work)

Have you felt stressed at your work in the last 12 months?	☐ No	☐ Yes, a bit	☐ Yes, a lot	☐ I'm no longer in the work force	☐ I'm out of work
How much do you now work compared with 12 months ago?	☐ I work more	☐ About the same	☐ I work less	☐ I'm no longer in the work force	☐ I'm out of work
If you work less now, is it because of your epilepsy?	☐ Yes	☐ Partly	☐ No		

Has your epilepsy put serious limitations on your life?

☐ No

☐ Yes, describe how: ____________________

How much alcohol do you drink on average in the course of a week? (Refers to beer, wine and spirits. If you drink less than 1 unit a week, write 0)

Write number of units: ____

Do you use recreational drugs? (f. ex. hash)

Put one tick	☐ Never	☐ Monthly	☐ Weekly	☐ Daily

The next questions are only relevant for women

Are you pregnant?

☐ Yes

☐ No

Do you plan to get pregnant within the next 12 months?

☐ Yes

☐ No

How much do you weight? (number of kg without clothes)

Write answer here: ☐☐☐

Have you driven a car in the last month?

- ☐ Yes
- ☐ No

What is your present need for contact with the outpatient clinic?

- ☐ I phone myself if I need to talk to someone
- ☐ I'd rather have someone phone me
- ☐ I'd like to have an outpatient appointment
- ☐ I don't know

Who has filled in this questionnaire?

- ☐ I have filled in the questionnaire
- ☐ I have had help filling in the questionnaire
- ☐ Someone else has filled in the questionnaire for me (f. ex., spouse, contact person)

May we phone you regarding your answers to the questionnaire?

- ☐ No
- ☐ Yes - and my phone number is: ☐☐☐☐☐☐☐☐

Here you can write a short note to the personnel that read the questionnaire
